# Sensor-Based Monitoring of Heart-Rate Responses to Ball Type in 9–11-Year-Old Tennis Players: A Preliminary Bayesian Repeated-Measures Study

**DOI:** 10.3390/s26144521

**Published:** 2026-07-16

**Authors:** Ana Piquer-Piquer, José María Giménez-Egido, José Francisco Guzmán, Miguel Crespo, Manrique Rodríguez-Campos, Rafael Martínez-Gallego

**Affiliations:** 1 Research Group on Sport Technique and Tactics, University of Valencia, 46010 Valencia, Spain; ana.piquer@uv.es (A.P.-P.); jose.f.guzman@uv.es (J.F.G.); rafael.martinez-gallego@uv.es (R.M.-G.); 2 Department of Physical Activity and Sport, University of Murcia, 30720 Murcia, Spain; josemaria.gimenez@um.es; 3 Development Department, International Tennis Federation, London SW15 5XZ, UK; miguel.crespo@itftennis.com

**Keywords:** internal load, IMU, young players, wearable sensors, biological maturation

## Abstract

**Highlights:**

**What are the main findings?**

- In this exploratory study, internal load (measured through maximum and average heart rate) provided anecdotal to moderate evidence in favor of the null model when switching between green and standard balls during short-format matches.
- Maturity-related indicators and physical activity level appeared more informative than ball type for explaining acute cardiac responses, although the strength of evidence differed across heart-rate outcomes.

**What are the implications of the main findings?**

- The transition from adapted to standard balls does not appear to increase acute cardiovascular demand, suggesting that young players may self-regulate their intensity.
- Training load and equipment transitions could be more effectively personalized based on the individual maturation profile and somatic age, rather than relying exclusively on chronological age categories.

**Abstract:**

Understanding internal load in youth tennis is essential for optimizing training strategies and ensuring healthy development. This study analyzed the influence of ball type (green vs. standard) on heart rate (HR) responses in 19 players (10.17 ± 1.1 years). Participants competed in 72 matches under a cross-over design while monitored with Wimu Pro™ Electronic Performance and Tracking Systems and Garmin HR bands. Data were analyzed using a Bayesian repeated-measures ANOVA, incorporating biological maturation and physical activity levels as covariates. Results indicated anecdotal evidence in favor of the null model for Max HR and moderate evidence in favor of the null model for Avg HR. These findings suggest no clear evidence of a meaningful ball-type effect on acute cardiovascular responses under the short-format match-play conditions studied. Individual characteristics, particularly maturity-related indicators and habitual physical activity, appeared more informative than ball type, although their evidential support differed across heart-rate outcomes. Therefore, the acute physiological response appeared to be similar between ball conditions in this specific pilot setting, but these findings should be interpreted as preliminary rather than definitive. Overall, these findings highlight the value of sensor-based monitoring for contextualizing internal load in youth tennis and suggest that equipment transitions should be guided by individual developmental and activity-related characteristics rather than ball type alone.

## 1. Introduction

Tennis is an intermittent sport characterized by short, high-intensity efforts followed by recovery periods [1]. It requires quick multidirectional movements, balance, and coordination, often played over long periods on different surfaces [2,3]. In youth tennis, comprehending the physiological demands and environmental constraints of youth tennis is crucial for optimizing training protocols and designing developmentally appropriate competitions that foster overall athletic progression [3]. Nowadays, the concept of load in sports includes both internal and external components [4]. Quantifying this load is vital to ensure it is high enough to improve performance but not so substantial that it causes injuries [5].

In recent years, the accurate monitoring of internal and external load in youth sports has been fundamentally transformed by the continuous advancement of wearable sensors and Electronic Performance and Tracking Systems (EPTS). These technological tools allow for the continuous, non-invasive assessment of physiological metrics within highly ecological, real-world competitive settings, effectively overcoming the logistical constraints of traditional laboratory testing. The critical role of sensor-based assessments in pediatric populations has been recently examined using perception–action technological devices in young athletes [6], highlighting the practical relevance of these modern tracking tools for accurately assessing neuromotor and performance-related variables. Previous research has analysed the physical demands on young athletes across various sports. In physical education, some authors compared internal and external load variations in 9-year-old students [7]. In youth soccer, studied U-19 players using Wimu Pro™ (RealTrack Systems, Almería, Spain) inertial devices to track external variables, such as total distance, high-intensity running, accelerations, and Player Load, alongside heart rate (HR) for internal load [8]. Similar studies in beach volleyball analysed physical demands in U-15 and U-19 players during competition, measuring relative distance and acceleration/deceleration actions [9].

Competitive youth tennis is defined by brief high-intensity periods (4–10 s) and short recoveries (10–20 s) [10]. HR is one of the most common indicators used to quantify internal load in these players [11,12]. Average HR values usually range between 60% and 80% of maximum heart rate (max HR), although intense rallies can push this above 95% [10,11] in junior and adult players. However, as these authors indicate, it is important to note that HR can sometimes overestimate physiological strain since it does not always perfectly match oxygen consumption variations during recovery. Players with better aerobic fitness can compete at lower relative intensities, which helps them recover faster and maintain technical efficiency [11]. Although, it is essential to distinguish these baselines from those of prepubertal athletes. 9–11-year-old players typically exhibit higher heart rate responses compared to adults due to their developing cardiovascular systems, smaller stroke volume, and higher sympathetic nervous system activity. Consequently, measuring HR specifically in this population is vital to ensure that internal load markers accurately reflect the physiological strain on a developing child, which may be underestimated if adult-derived benchmarks are applied.

When working with growing athletes, it is crucial to consider their biological maturation, as it affects physical performance [13]. Maturity status, often calculated through maturity offset, represents the time before or after the Peak Height Velocity (PHV). For 9–11-year-old players, maturity status is a more reliable predictor of performance than chronological age [14]. At this stage, children approach PHV, a critical period that can cause a temporary drop in neuromuscular performance and increased bilateral asymmetries due to rapid bone growth and coordination changes [14]. These changes make players more vulnerable to improper training loads, highlighting the need to monitor internal load [14,15]. PHV indicates the timing of maturation, representing the age of maximum growth speed during adolescence [16]. It is also important to evaluate the players’ sports participation level to understand how it relates to other physical characteristics [17,18].

Adapting equipment and the sporting environment to the needs of beginners is a key pedagogical principle for sustainable long-term development [19,20,21,22]. Material adaptation also has psychological benefits, allowing children to interact more efficiently with the game, which boosts motor learning and enjoyment [23,24,25]. From a biomechanical perspective, using adapted equipment in youth tennis helps reduce joint stress and prevent injuries during growth without compromising performance [26,27,28,29,30]. Studies show these adaptations improve technique, accuracy, and power, especially during early learning stages [27,31,32]. Coaches also value its impact on teaching and long-term participation [33,34,35,36].

In competition, low-compression balls and smaller courts lead to longer rallies and a more tactical game, like adult tennis [19,20,37,38,39,40,41,42,43,44,45,46]. Specifically, the green ball (25% less pressure than standard balls) is now mandatory for U-10 competitions by the ITF [47] and national federations like the RFET [48,49]. These balls are lighter and bounce more predictably, giving players more time to prepare and position themselves [31]. However, there is a lack of research analysing the physical demands and load on beginner players using these balls, especially in children aged 9–11 years, considering their biological maturity status. The main objective of this study is to analyse the internal load in 9–11-year-old tennis players, specifically examining how equipment modifications influence acute physiological responses to optimize training strategies and ensure healthy athletic development.

## 2. Materials and Methods

### 2.1. Sample

Nineteen players (14 boys and 5 girls) aged between 9 and 11 years (10.17 ± 1.1) played a total of 72 matches in an outdoor court, with an average duration of 21 min and 56 s (±8 min and 41 s). All players were competitive junior players, with an average of 3.63 ± 1.45 years of tennis practice and having competed in 7.65 ± 7.36 tournaments on average. The analytical dataset comprised repeated ecological match-observations obtained from the 19 players. Accordingly, the 72 valid match-observations represented repeated observations nested within players rather than independent participants. Detailed sample characteristics are provided in Table 1.

The players participated voluntarily, providing informed consent signed by their parents prior to the start of the tournament. Throughout the procedure, the ethical principles of the Declaration of Helsinki were followed, and approval of the protocol was obtained from the Ethics Committee of the University of Valencia (2025-FIS-3843453). This investigation is part of a broader, pre-planned research project designed to comprehensively evaluate the multi-dimensional load in youth competitive tennis players. To ensure a high level of analytical depth, the study was structured to analyze internal and external load variables as separate components. While a companion study focuses on external locomotor demands, such as total distance and accelerations, measured via EPTS, the present paper specifically targets the acute cardiovascular response.

### 2.2. Sample Size Considerations and Supplementary Fixed-N Bayes Factor Design Analysis

The present study was conducted in an ecological youth tennis tournament setting, where recruitment was constrained by the availability of competitive 9–11-year-old players, parental consent, and the logistical requirements of sensor-based monitoring during real match play. Therefore, the study was conceived as a preliminary pilot investigation aimed at generating ecologically valid evidence on heart-rate responses under two ball conditions.

To contextualize the operating characteristics of the available design, a supplementary fixed-N Bayes Factor Design Analysis was conducted. Bayes Factor Design Analysis is a simulation-based approach used to evaluate the expected evidential behavior of a research design when Bayes factors are used as the index of evidence. Rather than estimating classical statistical power, this approach quantifies the probability that a given design will yield different levels of Bayesian evidence, including evidence for H_0_, evidence for H_1_, inconclusive evidence, or evidence in the wrong direction under a specified data-generating scenario [50,51].

In the present study, this analysis focused specifically on the Green–Standard comparison, which represented the direct post hoc test of the ball-type effect. Because this comparison involved two repeated ball conditions, the Bayes Factor Design Analysis was approximated using a Bayesian paired-test framework with the same default Cauchy prior used in the post hoc comparisons, r = 1/√2. Monte Carlo simulations were conducted under a true null ball-type effect, that is, assuming no systematic difference in heart-rate response between green and standard balls.

For each simulated dataset, the Bayes factor was calculated and classified according to predefined evidential thresholds. BF_01_ > 3 was interpreted as moderate evidence for H_0_, BF_01_ > 10 as strong evidence for H_0_, BF_10_ > 3 as moderate evidence for H_1_, and values between these thresholds as inconclusive evidence. Evidence in the wrong direction was defined as obtaining BF_10_ > 3 under the simulated true null scenario.

The results of this supplementary fixed-N design analysis are presented in Table 2. This analysis was used to calibrate the evidential interpretation of the Green–Standard paired contrast. It was not used as a classical post hoc power analysis, nor as an inferential substitute for the Bayesian repeated-measures model.

This analysis indicated that, under a true null ball-type effect, the available Green–Standard paired contrast had a high probability of producing moderate evidence in favor of H_0_ and a low probability of producing moderate evidence in the wrong direction. Accordingly, the observed Bayes factors were interpreted using this simulation-based evidential benchmark.

### 2.3. Instruments and Data Collection

In order to register the internal load and performance of the players Electronic Performance and Tracking Systems (EPTS) and heart rate bands were used. Specifically, the Wimu Pro™ device (RealTrack Systems, Almería, Spain) was used, which is a hybrid EPTS integrating Local Positioning System (LPS) technology for indoor facilities and Global Navigation Satellite System (GNSS) technology for outdoor facilities as well as inertial sensors (IMU) (including accelerometers, gyroscopes, magnetometers and barometers) in a single unit [52], synchronized with Garmin (Garmin Ltd., Olathe, KS, USA) heart rate band. All data recordings were performed outdoors, using the GNSS system as a receiver with a sampling frequency of 18 Hz. The inertial sensors recorded data at a high-resolution sampling frequency of 1000 Hz. Heart rate data was collected with a Garmin chest strap with 4 Hz sampling rate through Ant+ technology.

Following data collection, a rigorous quality control protocol was applied to the GNSS and HR signals. First, an automatic detection algorithm within the SPRO™ software (version 2.2.2) flagged anomalies, such as sudden signal losses. Second, a visual inspection of the individual HR curves was conducted by the research team to manually identify and exclude non-physiological spikes or persistent dropouts caused by sensor displacement. From the total matches played, some were excluded from the final analysis if they met the following criteria: (i) loss of GNSS signal for more than 10% of the match duration, (ii) average number of satellites connected below 8, or (iii) consistent heart rate signal dropouts due to sensor displacement. Consequently, from 96 matches played, a final total of 72 valid match-observations was reached and utilized for the analysis to ensure the highest data integrity.

To control possible effects, some covariates were assessed, as the players’ level of physical activity through the PAQ-A scale [17], with a self-administered scale that has been widely used to assess the level of physical activity of schoolchildren over the previous 7 days. In addition, this scale has been validated in Spanish adolescents [18]. It consists of 8 items scored on a 5-point scale, with an extra item to determine any illness or situation that may have altered their physical activity during that week. To calculate the level of activity, only the average of the other 8 items is calculated. We acknowledge that the PAQ-C is the standard instrument for children in this age range. However, as the PAQ-A had been validated in Spanish and all the children were Spanish, we felt it would be more suitable. To mitigate potential inaccuracies in self-reporting due to the cognitive developmental stage of 9-to-10-year-olds, the questionnaire was administered under the direct supervision of the research team. Researchers were physically present and available to the players to clarify any conceptual doubts and assist them in interpreting the items, ensuring that the data collected accurately reflected their physical activity levels.

On the other hand, the maturity status of the players was calculated based on the variables of height and sitting height, following the validated formulas [16], which were used to obtain the maturity offset value, presented below:

- Girls: Maturity offset (years) = −7.709133 + (0.0042232 × (age × stature))
- Boys: Maturity offset (years) = −8.128741 + (0.0070346 × (age × sitting height))

### 2.4. Procedures

**Figure 1:**
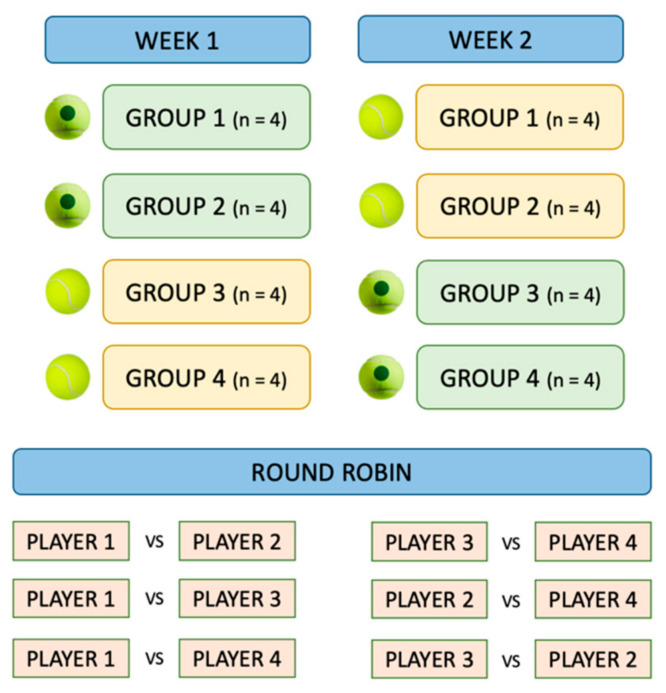
Competition design.

A cross-over design was used for the study, with four experimental groups of four players each, so that all participants were exposed to both conditions, allowing comparisons within the same groups in different situations. Participants were allocated to these groups based on their skill level to ensure balanced competitive levels within each round-robin group (see Figure 1). Due to the limited availability of official rankings in this age group, participants were allocated to these groups by certified coaches, based on skill level and years of practice. This stratification was intended to avoid mismatched pairings and guarantee representative physiological and locomotor demands during matches.

The matches were played in groups in a round-robin format, with one set of four games with a golden point, to avoid time differences that would excessively exceed the duration of the competition and the break times between matches. The competition took place on two different days, ensuring that all participants were exposed to both conditions, with two groups playing with the green ball (with 25% less pressure) and the other two groups playing with the standard ball, and the following week in reverse order. Each player played three matches per experimental condition. This ensured a sufficient volume of data to capture the physiological variability of each participant under both ball conditions. Although the initial design targeted 16 participants, three players from the first week were unable to attend the second session due to external scheduling conflicts. To preserve the ecological validity of the competition and maintain the strict four-player structure required for the tournament dynamics, three replacement players with matching skill levels, age profiles, and competitive experience were recruited for the second week. Consequently, the total sample reached 19 participants, and the number of valid match-observations ranged from 2 to 6 per participant. Due to external scheduling constraints, three players from the first session were unable to attend the second session. To preserve the ecological structure of the round-robin tournament and maintain comparable competitive conditions, three replacement players with similar age, skill level, and competitive experience were recruited for the second session. Consequently, the final dataset included 19 players and 72 valid match-observations. The Bayesian repeated-measures framework allowed all valid ecological match-observations to be retained while modelling subject-level dependency.

Prior to a 5 min on-court warm-up, the players’ height and seated height were measured to the nearest 0.1 cm with a tape measure on a flat vertical surface. For the standing height, participants stood barefoot with their heels, buttocks, and upper back in contact with the vertical surface and tape measure. For the sitting height, participants were seated on the floor with their back and buttocks touching the vertical backboard. The head was positioned in the Frankfort plane (orbital-tragion line horizontal), and traction was applied to the mastoid processes to ensure full spinal extension before measurement. These metrics were subsequently used to calculate maturity offset. The Wimu Pro™ unit was securely placed on the upper back, positioned between the scapulae, using a custom-fit vest provided by the manufacturer to minimize movement artifacts.

Lower-limb explosive capacity was assessed through Jump Height using the Countermovement Jump (CMJ) test [53]. Measurements were performed using a portable force. Before the test, players received a standardized demonstration and performed two submaximal practice trials to ensure proper technique. The protocol required participants to start from a standing position with their hands placed on their hips throughout the entire movement to eliminate the influence of arm swing and isolate lower-limb power. Upon a verbal signal, players performed a rapid downward countermovement to a self-selected depth, followed immediately by a maximal vertical thrust. Participants were instructed to land with their knees extended to ensure measurement accuracy. Each player performed three maximal trials, with a 60-s rest period between jumps to ensure full recovery. The highest jump (cm) was recorded and utilized as a covariate for the Bayesian model comparisons.

### 2.5. Variables Analysed

Dependent Variables:

- Max HR (bpm): maximal heart rate reached during the matches.
- Avg HR (bpm): average heart rate reached during the matches.

The analysis focused exclusively on absolute Maximum HR (Max HR) and Average HR (Avg HR), not including other traditional internal load indicators (time spent in specific HR zones or percentage of estimated HRmax). This decision was based on the fact that predictive HRmax equations exhibit high error margins in prepubertal populations. Furthermore, subjective metrics such as session-RPE were omitted due to the limited cognitive maturity of 9–11-year-olds to accurately interpret and anchor perceived exertion scales. Heart rate variability was also excluded, as it requires strict resting protocols that are incompatible with the tournament environment.

Independent Variable:

- Ball type: green ball (25% less pressure) versus standard ball [47,48].

Covariates:

Were considered as covariates the peak height velocity (PHV), estimated using the maturity offset equations [16]; general physical activity level, using the PAQ-A questionnaire [17,18]; years of tennis practice (Years of Practice) as an indicator of experience in the sport; jump height (Jump Height) as an indicator of explosive capacity; and the number of tournaments played (Tournaments). These covariates made it possible to control for possible effects of development, physical condition, and experience on the dependent variables.

Although match duration exhibited variability, it was not included as a covariate, based on the fact that Max HR and Avg HR are metrics of physiological intensity rather than volume; in intermittent sports like tennis, these values are primarily driven by the duration and intensity of individual rallies and subsequent recovery periods rather than the total match length. Furthermore, the use of a four-game set with a golden point was specifically implemented to minimize extreme time differences and limit the influence of cumulative fatigue on cardiac parameters.

### 2.6. Statistical Analysis

The statistical procedure began with an initial exploration of the data to test the assumptions of normality. To this end, the Shapiro–Wilk test was applied and the residuals were examined graphically using Q–Q plots; normality was assumed when the null hypothesis was not rejected and no systematic patterns of deviation were observed in the distributions. A base-10 logarithmic transformation was explored during preliminary diagnostics, but the final analyses were conducted and reported in the original raw scale (beats per minute, bpm) to facilitate physiological interpretation [54]. Prior to model estimation, multicollinearity among covariates was assessed using Variance Inflation Factors (VIF). All values were well below the commonly accepted threshold (VIF < 3), ranging between 1.20 and 1.60, indicating no evidence of multicollinearity and supporting the stability of the parameter estimates.

Next, descriptive statistics for the within-subject dependent variable were calculated: mean, standard deviation and 95% credible intervals (95% CrI). The inferential analysis was based on a Bayesian repeated-measures ANOVA. To account for the nested structure of the data, repeated match-observations were modelled within players by including subject-level random effects. This specification allowed ecological match-level information to be retained while avoiding the interpretation of match-observations as independent participants. The model included the Ball variable (levels: Green, Standard) as a within-subject factor [55].

The outcome variables were Max HR and Avg HR. The following covariates were included: PHV, PAQ-A, Years of Practice, Jump Height and Tournaments. Their impact was evaluated using a Bayesian model comparison approach, comparing alternative models against the null model based on Bayes Factors. Given the total number of possible combinations (64 models), only those with the strongest empirical support were retained according to the Bayes Factor (BF_10_), thereby prioritising the most empirically consistent structures. This approach aligns with a Bayesian model comparison framework, allowing identification of the most plausible models given the data.

Statistical evidence was expressed using Bayes’ factors for opposing hypotheses (H_0_ vs. H_1_), with BF_10_ interpreted as the evidence in favour of the alternative hypothesis and BF_01_ as its inverse [55,56,57]. The Bayes Factor was used as a continuous measure of evidence, which is linked to the quantifiable predictive performance of the models, also known as marginal likelihood [58]. The qualitative interpretation of BF_10_ followed the scale proposed by Lee & Wagenmakers [59], based on Jeffreys [60], which states:

- BF < 1/100: overwhelming evidence in favour of H_0_;
- 1/100–1/30: very strong evidence in favour of H_0_;
- 1/30–1/10: strong evidence in favour of H_0_;
- 1/10–1/3: moderate evidence in favour of H_0_;
- 1/3–1: anecdotal evidence in favour of H_0_;
- 1–3: anecdotal evidence in favour of H_1_;
- 3–10: moderate evidence in favour of H_1_;
- 10–30: strong evidence in favour of H_1_;
- 30–100: very strong evidence in favour of H_1_;
- BF > 100: extreme evidence in favour of H_1_.

In addition, Bayesian Inclusion Factors (BF incl) were calculated, which compare the fit of models containing a specific effect with their counterparts that do not include that effect. This index reflects the extent to which the posterior probabilities of an effect being present change relative to the prior probabilities after observing the data [55,61]. For interpretation, the standard criteria were applied: values > 3 support the inclusion of the effect, intermediate values (1/3, 3) are considered anecdotal, and values < 1/3 support its exclusion [62,63].

Both the BF_10_ and BF incl values were accompanied by the relative error (%) as an indicator of computational stability. In line with the methodological recommendations [55,64], errors of <3% were accepted, with estimates close to 0 (< 1%) being considered particularly robust. As an additional indicator of overall fit, the coefficient of determination (R^2^) was calculated, followed by a sensitivity analysis, restricting the comparison solely to those covariates that had shown favourable evidence for inclusion. The aim was to examine the robustness of the inferences under a reduced set of predictors [65]. This methodological strategy not only facilitates the interpretation of the results but also provides additional verification of the stability of the findings.

Regarding the Ball within-subject factor, Bayesian post hoc tests were applied between its two levels. As there were only two conditions, the Bayesian correction for multiple comparisons [66] was not applied. The contrasts were performed using Bayesian t-tests with a Cauchy prior distribution (0, r = 1/√2), and the resulting BF_10_ values were interpreted in accordance with the qualitative intervals described above. The supplementary fixed-N Bayes Factor Design Analysis described in the sample-size considerations section was used only to calibrate the evidential interpretation of the Green–Standard paired contrast. In line with previous BFDA frameworks [50,51], the analysis was interpreted as a design-level Monte Carlo procedure aimed at estimating the expected evidential behavior of the paired contrast under a specified data-generating scenario. Specifically, it quantified the probability that the available fixed-N contrast would yield moderate evidence for H_0_, strong evidence for H_0_, inconclusive evidence, or evidence in the wrong direction. Accordingly, this analysis was not treated as a classical power analysis or as an inferential substitute for the Bayesian repeated-measures model. The main inferential analyses were based on Bayesian repeated-measures model comparison, Bayesian Inclusion Factors, and Bayesian post hoc paired comparisons. The main Bayesian analyses were performed using the JASP software (version 0.95.0) (JASP Team, University of Amsterdam, Amsterdam, The Netherlands) [67]. The supplementary fixed-N Bayes Factor Design Analysis was performed using Monte Carlo simulations in RStudio (Posit Software, PBC, Boston, MA, USA). The presentation of the results followed current recommendations for reporting Bayesian analyses in applied sciences such as Physical Activity and Sport Sciences [55,68].

## 3. Results

The findings are presented by outcome variable. Model comparisons and evidence for effect inclusion are shown in tables, along with illustrations for descriptive and inferential comparisons by ball type.

### 3.1. Max HR (bpm)

Table 3 shows that the model with the highest relative support for Max HR included PHV as the only covariate (BF_10_ = 22.321), followed by the model including PHV and Years of Practice (BF_10_ = 14.662), and the model including Years of Practice alone (BF_10_ = 11.996). A fourth competitive model included PHV, PAQ-A, and Years of Practice (BF_10_ = 10.694). Overall, the BF_10_ values for the top-ranked models indicated moderate to strong evidence in favour of these models relative to the null model. The relative error for the best-supported model (PHV) was 1.590%. The model-averaged coefficient of determination was R^2^ = 0.458 (CrI95% = [0.275, 0.597]). A sensitivity analysis including covariates with BF_incl_ > 1 (PHV, PAQ-A, and Jump Height) showed a moderate R^2^ of 0.452 [0.264, 0.594], maintaining model fit even with lower complexity (Table 3).

The effect inclusion analysis in Table 4 showed strong evidence in favour of PAQ-A (BF_incl_ = 15.842; P(incl|data) = 0.941) and moderate evidence in favour of PHV (BF_incl_ = 4.985; P(incl|data) = 0.833). Jump Height showed only anecdotal evidence for inclusion (BF_incl_ = 1.451), whereas Ball, Years of Practice, and Tournaments presented BF_incl_ values below 1, indicating that the data did not support their inclusion in the model. The model-averaged posterior summary indicated a stable intercept (M = 178.019, SD = 2.014; 95% CrI [173.749, 182.023]). The effects of PHV (M = 4.667, 95% CrI [0.108, 8.933]) and PAQ-A (M = 11.695, 95% CrI [2.389, 20.925]) showed credible intervals that excluded zero, indicating evidence of positive effects on Max HR. In contrast, the remaining predictors presented 95% CrIs that included zero, reflecting greater uncertainty regarding their specific contributions.

**Figure 2:**
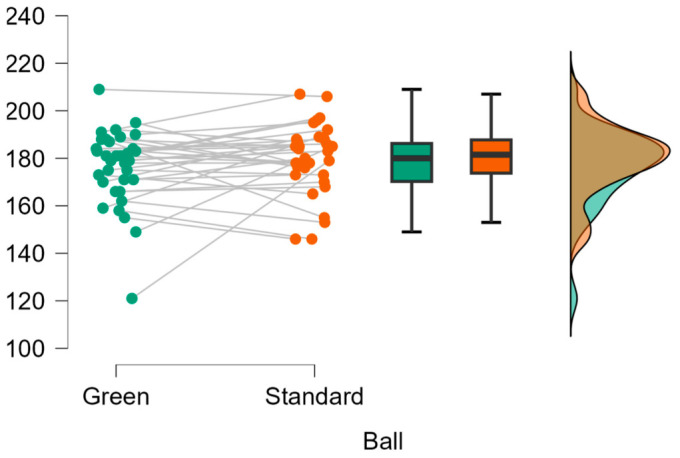
Descriptive analysis of Max HR according to game ball.

Regarding the “Ball” factor, descriptive means per condition were (Figure 2): Green (M = 176.3, SD = 15.92, 95% CrI = [170.8, 181.9]) and Standard (M = 179.7, SD = 14.58, 95% CrI = [174.6, 184.8]). The post hoc comparison showed a BF_10_,U = 0.364, which represents anecdotal evidence in favor of the null hypothesis. In short, the data are approximately 2.7 times more likely under the null hypothesis than under the alternative hypothesis.

### 3.2. Avg HR (bpm)

The comparison between the null and alternative models in Table 5 showed that the model with the most support included the covariates PHV (BF_10_ = 1.641), followed by PHV + Years of Practice (BF_10_ = 1.412) and Years of Practice (BF_10_ = 1.171). Overall, BF_10_ values were close to 1, indicating only anecdotal evidence in favor of models with predictor variables. The relative error for models with BF_10_ > 1 was less than 1%. The model-averaged coefficient of determination was R^2^ = 0.382 (CrI95% = [0.241, 0.528]). Accordingly, the sensitivity analysis showed an R^2^ = 0.382 (CrI 95% = [0.231, 0.526]), when including only the PAQ-A covariate, which was the only one with BF_incl_ > 1 (Table 5). Since both coefficients are similar, the more complex model can be considered robust.

The effect inclusion analysis in Table 6 showed anecdotal evidence in favour of PAQ-A (BF_incl_ = 2.159; P(incl|data) = 0.683), while PHV presented evidence close to neutrality (BF_incl_ = 0.918; P(incl|data) = 0.479). The remaining predictors, including Ball, Jump Height, Years of Practice, and Tournaments, showed BF_incl_ l values below 1, indicating evidence against their inclusion in the model. The model-averaged posterior summary indicated a stable intercept (M = 147.120, SD = 1.968; 95% CrI = [142.999, 150.771]). Although PAQ-A showed a positive mean effect and PHV a smaller positive effect, their 95% credible intervals included zero (PAQ-A: [−1.249, 14.113]; PHV: [−1.872, 6.278]), indicating substantial uncertainty regarding their contribution. Similarly, the remaining predictors showed mean effects close to zero, with wide credible intervals including zero, further supporting the lack of consistent evidence for their influence on Avg HR.

**Figure 3:**
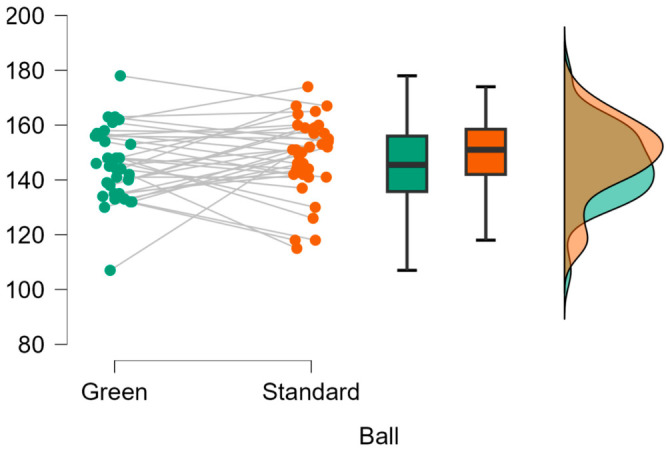
Descriptive analysis of Avg HR according to game ball.

Figure 3 shows mean values and overlapping credible intervals when comparing Avg HR by ball type: Green (M = 145.8, SD = 13.33; 95% CrI = [141.2, 150.5]) and Standard (M = 148.4, SD = 14.38; 95% CrI = [143.4, 153.4]). Pairwise comparisons between conditions resulted in a BF_10_,U = 0.278, indicating moderate evidence in favor of the null hypothesis. In practical terms, the data are approximately 3.6 times more likely to support the absence of differences between ball types than the alternative.

## 4. Discussion

The main objective of this preliminary pilot study was to analyse the internal load in 9–11-year-old tennis players and determine how equipment modifications influence their acute physiological response. The primary findings indicate an absence of a credible effect of ball type (green vs. standard) on internal load, with the data providing anecdotal to moderate evidence in favor of the null model, measured through maximum heart rate (Max HR) and average heart rate (HR avg). Instead, individual characteristics, particularly maturity-related status and habitual physical activity, appeared more informative than ball type, although the strength of evidence differed across heart-rate outcomes. Although the literature emphasizes the importance of adapting equipment to improve technique and prolong rallies, our preliminary results show a lack of evidence for including the “Ball” factor in the explanatory models for internal load. Max HR averages were similar between the green ball (176.3 bpm) and the standard ball (179.7 bpm), with a Bayes factor (BF10,U = 0.364) supporting the null hypothesis. This suggests that, within the context of short round-robin matches, the acute cardiovascular load is similar regardless of ball compression. This could be because perceived effort and the intensity of intermittent play remain constant despite variations in ball speed. However, it is important to note that our study assessed acute heart rate responses and did not directly measure cumulative fatigue, recovery kinetics, perceived exertion, or blood lactate. Therefore, while cardiovascular demand appears stable, broader fatigue-related or neuromuscular effects cannot be entirely excluded.

The supplementary fixed-N Bayes Factor Design Analysis provides a simulation-based benchmark for interpreting the Green–Standard paired contrast. Under a true null ball-type effect, the available paired contrast showed a high probability of yielding moderate evidence in favor of H_0_ and a low probability of yielding moderate evidence in the wrong direction. This benchmark is consistent with the observed post hoc Bayes factors, which provided anecdotal evidence for Max HR and moderate evidence for Avg HR in favor of H_0_. Therefore, the ball-type findings should be interpreted as calibrated evidence against a clear acute cardiovascular effect under the present ecological match-play conditions, rather than as definitive proof of equivalence between ball conditions.

A relevant contribution of this exploratory work is that individual characteristics appeared more informative than ball compression for explaining heart-rate variability., surpassing the type of material used. This result is consistent with literature indicating that players with a higher biological age (post-PHV) achieve better results in almost all variables of speed, agility, and explosive power compared to their pre-PHV peers [69]. Specifically, it has been documented that players with a more advanced maturity status present a 16% to 27% advantage in vertical jump and a 5% to 8% advantage in linear speed and change in direction [69]. Our data suggest that players closer to their PHV record reduced active time and a lower work-to-rest ratio [70]. This seemingly contradictory phenomenon is explained by the profound morphological and neural adaptations occurring during the biological transition: increased muscle mass, stride length, and force-generating capacity allow for greater movement economy [69,71]. Therefore, the physical load perceived on the court depends more on the biomechanical efficiency of the developing athlete than on the physical properties of the ball [14,72].

The observed absence of a credible effect on HR between green and standard balls challenges the pedagogical assumption that adapted equipment inherently reduces cardiovascular load in prepubertal players. Although low-compression balls facilitate longer exchanges and greater participation, 9–11-year-old players seem to apply a self-regulation mechanism for effort [25,31]. It is plausible that young tennis players unconsciously adjust their movement intensity to the affordances offered by the equipment to maintain a sustainable and competitive effort level [70]. This behaviour is typical of the intermittent nature of tennis, where total load is a product of both the duration and intensity of high-intensity efforts. In this context, studies have shown that fatigue critically affects technical precision (up to 81%) and kinematic efficiency before affecting average cardiac parameters [1,73]. In fact, fatigue induces variability in motor control and reduces the efficiency of the kinetic chain in the serve, affecting ball speed and trunk stability [73,74].

A critical finding was the positive influence of PAQ-A and PHV on Max HR. The model with the most relative support for max HR included PHV and physical activity level, with clear evidence in favor of their inclusion (BF_incl_ = 15.842 for PAQ-A and 4.985 for PHV). The positive relationship between PHV and internal load reinforces the idea that physiological analysis at these ages must be linked to maturity status rather than chronological age. Since children at this stage are approaching their peak height velocity, their vulnerability to training loads increases due to neuromuscular and coordination changes [71].

The Bayesian models developed in this study reached model-averaged coefficients of determination of R^2^ = 0.458 for Max HR and R^2^ = 0.382 for Avg HR. These results indicate that while biological maturation and physical activity levels are strong predictors, approximately 54% to 62% of the heart rate variability remains unexplained by the included covariates. This finding suggests that internal load in 9–11-year-old tennis is a multi-factorial phenomenon influenced by variables beyond individual characteristics, emphasizing the need to interpret these exploratory data cautiously given the modest sample size. Factors such as ambient temperature and humidity, which are critical for understanding environmental physiological demands, as well as situational variables like match outcome, emotional stress, or the tactical level of the opponent, likely account for the remaining variance.

From a professional and ethical perspective, our findings highlight the need for training program designs to transcend chronological age. Coaches must understand that physiological stress and load responsiveness are intrinsically linked to individual development and the child’s active lifestyle. This individualized approach is strongly supported by recent advancements in sports technology. The application of wearable and perception-action technological devices in youth sports provides meaningful, objective data on performance variables that transcend standard age categories [6]. By adopting such sensor-based assessment tools, practitioners can effectively tailor training and monitor loads according to the athlete’s specific biological profile rather than relying exclusively on standardized chronological brackets.

The transition from the green ball to the standard ball should not be a bureaucratic event based on the athlete’s birthday, but a process guided by their biological profile and technical-tactical mastery. Using Maturity Offset allows coaches to identify periods of adolescent awkwardness, where disproportionate growth alters motor coordination and joint stiffness, increasing injury risk [13,14]. During these phases, using green balls allows the player to continue developing tactical intelligence and efficacy without overloading a neuromuscular system that is undergoing full restructuring [70,75]. Likewise, the Youth Physical Development (YPD) Model suggests that while strength and speed are trainable at all stages, the transition to more demanding materials should align with the natural increase in post-PHV androgenic hormone levels, which provide the structural support needed to handle higher-pressure balls without compromising hitting mechanics [71].

### Limitations and Future Research Lines

Despite the contributions of this study, several limitations must be acknowledged. First, several specific limitations regarding the sample must be acknowledged. First, the unequal sex distribution within our cohort may limit the generalizability of the findings across genders. Second, biological maturation was estimated indirectly using the Maturity Offset method rather than direct skeletal age assessments due to lack of instrumentation. Given these constraints, the small participant sample size and the nested nature of the match observations, the findings of this study should be interpreted as exploratory. Future research should aim to replicate these models using larger, sex-balanced cohorts and direct maturational assessments.

The generalizability of the findings is also constrained by the competitive format used. The matches followed a round-robin structure consisting of a single 4-game set with a “golden point”. While this format was chosen to control duration and ensure representative demands, the physiological responses might differ in standard competitive formats. An additional methodological limitation is the replacement of three participants between testing sessions to maintain the organizational structure of the tournament. While necessary for ecological validity, this substitution disrupted the perfect repeated-measures structure and resulted in an unbalanced dataset, although our Bayesian analytical framework effectively mitigated the loss of statistical power.

Furthermore, regarding the assessment of physical activity, the PAQ-A questionnaire was originally validated for adolescents. Although it is widely used in school settings, its application in 9-to-10-year-old children may present challenges in self-reporting accuracy due to the cognitive developmental stage of this specific age group. This was minimized through the presence of two authors during the questionnaire filling for answering doubts.

The Bayesian models developed in this study yielded moderate model-averaged coefficients of determination. This emphasizes that internal load in Under-10 tennis is a highly complex, multi-factorial phenomenon. Unmeasured factors such as environmental conditions (temperature and humidity) and situational variables (match outcome, opponent’s tactical level) likely account for this remaining variance, highlighting the need for future explanatory models to integrate these dimensions.

Additionally, internal load was monitored exclusively through heart rate. Although it is considered a gold standard, future research should incorporate HR variability to evaluate autonomic recovery, as well as Rating of Perceived Exertion scales adapted for children to capture the psychological dimension of fatigue; or recovery kinetics and lactate. Finally, the study focused on a specific age range; expanding future research toward earlier stages (red/orange balls) or later phases would provide a longitudinal perspective on how biomechanical efficiency and maturation mitigate the impact of standard equipment.

## 5. Conclusions

The preliminary findings of this exploratory study suggest that, under the specific conditions of this study, the acute physiological response in 9–11-year-old tennis players measured through internal load, appears to be similar regardless of the compression of the ball used (green vs. standard). The Bayesian analysis provided evidence consistent with the absence of a meaningful difference in heart rate in this experimental setting, suggesting that players in developmental stages may possess self-regulation mechanisms that allow them to adapt their movement intensity to the mechanical properties of the equipment, maintaining a stable and functional level of cardiovascular stress. Therefore, the transition from low-compression balls to standard balls does not appear to represent, by itself, an increased risk of excessive metabolic fatigue in this age group during short competitive matches.

However, this study highlights that the design of training loads and competitive progression should ideally go beyond the athletes’ chronological age. Identifying PHV and previous physical activity levels as the most robust predictors of cardiac response in our models suggests that these variables are more informative predictors of internal load than the equipment itself. Consequently, sensor-based monitoring in youth tennis gains its true clinical and sporting value when internal load data are normalized according to the individual maturation profile, allowing for early detection of periods of coordination vulnerability or adolescent awkwardness.

Finally, this work reinforces the importance of integrating active lifestyle habits as a foundation for on-court performance. High levels of general physical activity not only led to a greater capacity for effort during competition but also appeared to act as a protective factor against elevated cardiovascular strain. In terms of practical application, and pending validation in larger cohorts, it is recommended that coaches and federations treat the transition of materials as a flexible and personalized process, guided by the player’s somatic maturity and mechanical efficiency, thus ensuring the healthy, long-term development of the young tennis player.

## Figures and Tables

**Table 1:** Sample description.

	Total (*n* = 19)		Girls (*n* = 5)		Boys (*n* = 14)	
	Mean	S. D.	Mean	S. D.	Mean	S. D.
Age (years)	10.17	1.10	10.5	0.69	10.8	1.23
Height (cm)	139.6	8.6	137.0	4.8	139.6	8.6
Sitting height (cm)	69.96	5.46	70.9	2.51	69.27	5.46
Maturity offset (years)	−2.84	0.98	−2.24	0.87	−3.14	0.84
Peak height velocity (PHV) (years)	12.94	0.95	12.11	0.48	13.26	0.66
Physical Activity Questionnaire (PAQ-A)	3.49	0.57	2.82	0.49	3.77	0.33
Experience in tennis practice (years)	3.63	1.45	4.30	1.20	3.39	1.50
Tournaments played (*n*)	7.65	7.36	2.4	2.51	9.8	5.34
Weekly training hours (*n*)	6.0	2.9	4.8	1.64	6.4	3.17

**Table 2:** Supplementary fixed-N Bayes Factor Design Analysis for the Green–Standard paired contrast under a true null ball-type effect.

Evidential Outcome	Criterion	Probability (%)	Interpretation
Moderate evidence for H_0_	BF_01_ > 3	73.7	The paired contrast had a high probability of yielding moderate evidence for H_0_ under a true null ball-type effect.
Strong evidence for H_0_	BF_01_ > 10	0.0	The paired contrast was not expected to yield strong evidence for H_0_ under the specified design and prior.
Misleading moderate evidence for H_1_	BF_10_ > 3	1.5	The probability of obtaining misleading evidence for a ball-type effect under H_0_ was low.
Inconclusive evidence	1/3 ≤ BF_10_ ≤ 3	24.8	Approximately one quarter of comparable simulations would be expected to remain inconclusive.

Note. BF_01_ = evidence in favor of the null hypothesis over the alternative hypothesis; BF_10_ = evidence in favor of the alternative hypothesis over the null hypothesis. The analysis was approximated using a Bayesian paired-test framework with a default Cauchy prior, r = 1/√2, under a simulated true null ball-type effect.

**Table 3:** Selection of the top five Bayesians models with random effects per subject for predicting Max HR.

	P(M)	P(M|Data)	BFM	BF_10_	Error %
Null model including subject-level random effects	0.016	0.007	0.428	1.000	
(PHV)	0.016	0.151	11.170	22.321	1.590
PHV + Years of Practice	0.016	0.099	6.916	14.662	1.584
Years of Practice	0.016	0.081	5.548	11.996	1.262
PHV + PAQ-A + Years of Practice	0.016	0.072	4.899	10.694	1.840

Note: P(M) = Prior model probability (probability of the model before observing the data); P(M|data) = Posterior model probability (probability of the model after observing the data); BFM = Posterior model odds; BF_10_ = Bayes Factor quantifying the evidence for the specific model relative to the null model; error % = relative computational error of the estimate.

**Table 4:** Inclusion evidence and credible intervals from the model-averaged posterior summary in the Bayesian repeated-measures ANOVA for Max HR predictors.

		Mean 95%|CrI Posterior	P(Incl|Data)	P(Excl|Data)	BF_incl_
Ball	Green	−1.55 [−4.143, 1.036]	0.325	0.675	0.481
	Stand.	1.41 [−1.125, 3.944]			
PHV		4.67 [0.108, 8.933]	0.833	0.167	4.985
PAQ-A		11.70 [2.389, 20.925]	0.941	0.059	15.842
Jump Height		−1.35 [−2.966, 0.274]	0.592	0.408	1.451
Years of Practice		0.65 [−2.112, 3.416]	0.349	0.651	0.535
Tournaments		−0.07 [−0.603, 0.469]	0.314	0.686	0.458

Note: 95% CrI = 95% Credible Interval (the range within which the true parameter value falls with a 95% probability). P(incl|data) = Posterior inclusion probability (the probability that the predictor is included in the model after observing the data). P(excl|data) = Posterior exclusion probability. BF_incl_ = Bayesian Inclusion Factor (quantifies the evidence for including a predictor over excluding it).

**Table 5:** Selection of the top five Bayesians models with random effects per subject for predicting Avg HR (bpm).

	P(M)	P(M|Data)	BFM	BF10	Error %
Null model including subject-level random effects	0.016	0.044	2.888	1.000	
(PHV)	0.016	0.072	4.883	1.641	0.927
(PHV) + Years of Practice	0.016	0.062	4.157	1.412	0.480
Years of Practice	0.016	0.051	3.409	1.171	1.362
(PHV) + PAQ-A + Years of Practice	0.016	0.041	2.700	0.937	0.571

Note: P(M) = Prior model probability (probability of the model before observing the data); P(M|data) = Posterior model probability (probability of the model after observing the data); BFM = Posterior model odds; BF_10_ = Bayes Factor quantifying the evidence for the specific model relative to the null model; error % = relative computational error of the estimate.

**Table 6:** Inclusion evidence and credible intervals from the model-averaged posterior summary in the Bayesian repeated-measures ANOVA for Avg HR predictors.

		Mean|95% CrI	P(Incl|Data)	P(Excl|Data)	BF_incl_
Ball	Green	−1.099 [−3.625, 1.303]	0.271	0.729	0.372
	Stand.	1.099 [−1.386, 3.590]			
(PHV)		2.111 [−1.872, 6.278]	0.479	0.521	0.918
PAQ-A		6.184 [−1.249, 14.113]	0.683	0.317	2.159
Jump Height		−0.551 [−2.096, 0.884]	0.394	0.606	0.650
Years of Practice		−0.709 [−3.248, 1.745]	0.375	0.625	0.600
Tournaments		0.079 [−0.468, 0.507]	0.341	0.659	0.517

Note: 95% CrI = 95% Credible Interval (the range within which the true parameter value falls with a 95% probability). P(incl|data) = Posterior inclusion probability (the probability that the predictor is included in the model after observing the data). P(excl|data) = Posterior exclusion probability. BF_{incl} = Bayesian Inclusion Factor (quantifies the evidence for including a predictor over excluding it).

## Data Availability

The data presented in this study are available on request from the corresponding author. The data are not publicly available due to privacy and ethical restrictions, as the study involved the physiological monitoring of minors and was conducted under the confidentiality protocols.

## Abbreviations

The following abbreviations are used in this manuscript:

Max/Avg HR	Maximal Heart Rate/Average Heart Rate
PHV	Peak Height Velocity
U19, U12	Players under the indicated age (under 19-year-old, under 12…)
RFET	Real Federación Española de Tenis
ITF	International Tennis Federation
BF	Bayes Factor
BF_incl_	Bayes inclusion factors
CrI	Credible interval
cnt	Count
s	Seconds
